# Cognition in Males and Females with Autism: Similarities and Differences

**DOI:** 10.1371/journal.pone.0047198

**Published:** 2012-10-17

**Authors:** Meng-Chuan Lai, Michael V. Lombardo, Amber N. V. Ruigrok, Bhismadev Chakrabarti, Sally J. Wheelwright, Bonnie Auyeung, Carrie Allison, MRC AIMS Consortium, Simon Baron-Cohen

**Affiliations:** 1 Autism Research Centre, Department of Psychiatry, University of Cambridge, Cambridge, United Kingdom; 2 Centre for Integrative Neuroscience and Neurodynamics, School of Psychology and Clinical Language Sciences, University of Reading, Reading, United Kingdom; University of Western Brittany, France

## Abstract

The male bias in autism spectrum conditions (ASC) has led to females with ASC being under-researched. This lack of attention to females could hide variability due to sex that may explain some of the heterogeneity within ASC. In this study we investigate four key cognitive domains (mentalizing and emotion perception, executive function, perceptual attention to detail, and motor function) in ASC, to test for similarities and differences between males and females with and without ASC (n = 128 adults; n = 32 per group). In the mentalizing and facial emotion perception domain, males and females with ASC showed similar deficits compared to neurotypical controls. However, in attention to detail and dexterity involving executive function, although males with ASC showed poorer performance relative to neurotypical males, females with ASC performed comparably to neurotypical females. We conclude that performance in the social-cognitive domain is equally impaired in male and female adults with ASC. However, in specific non-social cognitive domains, performance within ASC depends on sex. This suggests that in specific domains, cognitive profiles in ASC are modulated by sex.

## Introduction

Autism spectrum conditions (ASC) affect more males than females, with a ratio of 4.3 to 1 reported in earlier studies [1]. This male bias has led to females with ASC being under-studied. This neglect may have implications not only for our understanding of ASC but also for clinical practice and how we diagnose ASC. For example, it could be that the male bias in ASC is partly due to the current diagnostic criteria being more aligned to presentations that are more apparent in males. In other words, we may be under-diagnosing females due to their ‘non-male-typical’ profile, or their ability to better camouflage/compensate for their difficulties [2]–[9].

Recent epidemiological surveys have reported a lower male bias (2.0–2.6∶1) in the prevalence of ASC in the general population [10]–[12], even among those who are high-functioning (1.7∶1) [10]. A recent prospective report of infant-siblings at high-risk for developing autism showed only a small (1.65∶1) male bias in those later diagnosed at 3 years of age, and that a large proportion of individuals accounting for this reduction in male bias were females at higher-functioning levels [13]. These lines of evidence suggest increasing awareness of females on the autistic spectrum, especially of those without intellectual disability. There is thus an immediate need for better understanding of females with ASC [14], [15], particularly with respect to their similarities and differences compared to males with ASC.

Previous studies of sex differences in core behavioral features of autism are inconsistent, in samples that range widely in age and IQ. A few studies find no sex differences in core features [16]–[19], but others do show sex differences [15], [20]–[23]. At the biological level however, females with ASC show different profiles in serum biomarkers [24], genetics [25]–[27] neuroanatomy [28], and early brain overgrowth [29]–[32]. However, we still know little about cognition, with the exception of a small number of studies showing possible differences in executive function and visuospatial processing. For example, Nydén and colleagues reported that, in a clinic sample, girls (mean age 9.8 years, 5 with ASC, 12 with ADHD) performed worse than boys (mean age 10.1 years, 5 with ASC, 12 with ADHD) on the Tower of London test (measuring planning ability) [33]. Koyama and colleagues found that on a standardized IQ test, girls (mean age 8.2 years) with high-functioning ASC performed better than boys (mean age 9 years) with high-functioning ASC on processing speed, coding and symbol search, but that boys were better on block design [34]. Bölte and colleagues found that adolescent females with high-functioning ASC outperformed males with high-functioning ASC on the Trail Making Test (measuring set-shifting), but the opposite was observed in their unaffected siblings [35]. Lemon et al. reported that girls (aged 6 to 16 years) with high-functioning ASC performed worse than typical girls on a stop signal task (measuring response inhibition), but boys with high-functioning ASC performed comparably to typically developing boys and girls, who did equally well [36]. The present study extends our earlier report of the *behavior* of high-functioning adult males and females with ASC [15] to the level of *cognition*.

Cognitive features of ASC range from differences in basic sensory perceptual processing to high-level complex social cognition [37]. One way to illuminate the full picture is to apply a wide array of cognitive tasks [38], [39]. As one of the first studies to formally compare adult males and females with and without ASC we opt for a more parsimonious approach, selecting the most widely used tasks in four key domains of cognition closely tied to ASC characteristics: (i) mentalizing and emotion perception, (ii) executive function, (iii) perceptual attention to detail, and (iv) motor function. Based on the emerging evidence of potential sex differences within autism, we test if the effect of autism is dependent on sex within each of these cognitive domains. If confirmed, this would be expressed as a significant sex-by-diagnosis interaction in a two-way factorial design (factor 1: sex, factor 2: diagnosis).

## Materials and Methods

### Ethics Statement

Informed written consent was obtained for all participants in accordance with procedures approved by the Suffolk Research Ethics Committee.

### Participants

Thirty-three neurotypical male, 35 neurotypical female, 45 ASC male and 38 ASC female right-handed Caucasian English-speaking adults were recruited through the UK Medical Research Council Autism Imaging Multicentre Study (MRC AIMS) consortium (full details are described elsewhere [15]). The inclusion criteria included being aged between 18 to 49 years, without intellectual disability (IQ ≥70), and participants in the ASC group had a formal clinical diagnoses of autistic disorder or Asperger’s disorder, based on DSM-IV [40] or ICD-10 [41] criteria, from a psychiatrist or clinical psychologist in the UK National Health Service.

Exclusion criteria (for all groups) included a history of or a current psychotic disorder, substance-use disorder, severe head injury, syndromic genetic disorder associated with autism (e.g. fragile X syndrome, tuberous sclerosis), intellectual disability (i.e., IQ <70), hyperkinetic disorder, Tourette’s disorder, any other medical condition significantly affecting brain function (e.g. epilepsy), and/or current use of antipsychotic medication. The neurotypical groups did not have an ASC diagnosis themselves nor was it present in their family history.

The ASC participants were further selected based on their ADI-R scores [42]. To be included, they had to reach the diagnostic algorithm cut-offs for ‘autism’ but were permitted to score one point below threshold in one of the three core symptom domains, to allow for possible underestimation of early developmentally atypical behaviours in the recall of caregivers whose children were now adults; these all followed our earlier studies and rationale for inclusion [15], [43]–[46]. Thirty-three male and 29 female adults scored above the threshold. Another three women, although not having ADI-R data available (because their childhood caregivers were not available to be interviewed), were also included for the following reasons: One scored above the cut-off for ‘autism spectrum’ on the Autism Diagnostic Observation Schedule (ADOS) [47], one previously received a diagnosis using the Adult Asperger Assessment (AAA) [48] which had incorporated care-giver reports on childhood behaviors, and one had been diagnosed by an expert clinician with assessments that included a comprehensive childhood developmental history. After matching for age, IQ and sample size across groups, the final cohort for analysis consisted of 32 participants per group.

### Measures

#### Mentalizing and emotion perception

Impaired social-emotional-communication is the cardinal feature of ASC [37], [49], [50]. This can be viewed as stemming from two different aspects of atypical functioning: secondary and primary intersubjectivity [51]. For cognition related to secondary intersubjectivity, theory of mind (ToM) or mentalizing deficits have been found across the life span, from the classical first-order ToM deficit in children [52], to complex ToM deficits revealed in moral judgments [53] and spontaneous ToM [54] in adults with ASC. Here we used the ‘Reading the Mind in the Eyes’ test (Eyes Test) [55] to investigate mentalizing ability. The Eyes Test, comprising 36 items, requires the individual to infer mental status solely from the information of a person’s eyes and the immediate surrounding areas of the face in gray-scale photographs. Both the correct score and reaction time (RT) were taken as outcome measures. However, due to its high cognitive demands, total correct score was considered more informative. RT was positively skewed; and for parametric methods to be applicable it was log-transformed to approximate a normal distribution.

Cognition related to primary intersubjectivity involves processes supporting dyadic interaction such as face processing, emotion perception and social motivation. In particular, facial emotion recognition is frequently reported to be atypical in ASC. For example, adolescents and adults with ASC demonstrate atypical processing of basic negative facial emotions [56]–[58], subtle expressions of fear [59], sadness [60], disgust [61], as well as more complex emotional states [62]. In this study, an online version of the Karolinska Directed Emotional Faces Test (KDEF Test) was used to measure basic emotion perception. The KDEF Test is a 140-item basic emotion recognition task using stimuli from the KDEF database of photographs of basic emotions [63], comprising seven sets of 20 color faces presenting six basic emotions (happy, sad, angry, fear, disgusted, surprised) and a neutral expression, with stimuli presented in random order. Participants were asked to choose one from seven responses to identify the emotion of the face stimuli, by pressing a key from 1–7 on the keyboard, and were instructed to go as quickly and as accurately as possible. Due to potential ceiling effects in accuracy, we opted for reaction time but calculated *accuracy-adjusted reaction time* (aaRT = mean reaction time/accuracy) for each emotion, to take into account performance information on both [64]. The seven aaRTs were all positively skewed and were therefore log-transformed to approximate a normal distribution. Owing to the presence of a small number of right-tail outliers even in these log-transformed aaRTs, *winsorizing* was further performed as a trial by recoding all outliers to the score that fell on two standard deviations above the mean. [Note: Outliers (all/extreme) were identified by each group. There were in total 0/0 for happy, 2/0 for sad, 4/3 for angry, 5/1 for fear, 5/1 for disgusted, 6/2 for surprised and 3/0 for neutral faces. There was no individual who was an outlier on all or most emotions. This indicated that the outlier was poor in recognizing particular emotion(s) but not generally slow/inaccurate.] This procedure, however, did not change any of the outcomes of statistical comparisons so results from the non-winsorized aaRTs will be reported. The outlier aaRT contains information of the emotion recognition ability on a specific emotion but not generally all emotions, reflecting the participant’s actual ability on a particular emotion. Winsorizing may reduce the extent of violation to assumptions for parametric tests, but as a consequence the information about particularly poor performance on a specific emotion, which is very informative for group comparisons, may be lost. We therefore report the results from the non-winsorized data. This is also because the F-test is robust to violations to assumptions [65].

#### Executive function

*Executive dysfunction* is a (non-specific) feature of ASC [66]–[72]. Many aspects of executive function, including *planning*, *set-shifting*, *inhibition*, *generativity* and *self-monitoring*, have been reported as impaired in people with ASC [73]. However there is inconsistency associated with experimental designs, IQ and co-occurring conditions such as hyperkinetic disorder or Tourette’s syndrome [74]. Relatively consistent findings point to difficulties in planning, set-shifting and inhibition of a prepotent response [75].

In the present study we used an online version of the Go/No-Go task to test inhibition and signal detection. Participants were instructed to press the ‘left key’ (‘Q’ on the left side of the keyboard) using the left hand when seeing a bold arrow pointing to the left presented on the screen, the ‘right key’ (‘P’ on the right side of the keyboard) using the right hand when seeing an arrow pointing to the right, and to not respond when seeing an arrow pointing upward. A total of 300 stimuli (110 left, 110 right, and 80 upward arrows) were presented randomly. Reaction time and responses for all 300 items were recorded. Results were first explored by calculating the classic *commission error* (pressing ‘left’ or ‘right’ when the stimuli is upward and should be ignored) and *omission error* (making no response when ‘left’ or ‘right’ should be pressed). Due to the highly skewed distribution, these error rates were rank-transformed to approximate a normal distribution prior to analysis. The performance was then re-analyzed within the framework of signal detection theory (SDT) [76] to estimate two major parameters: *sensitivity* (*d’* = *Z_Hit_* – *Z_FA_*, where *Z_Hit_* is the corresponding *Z* value in the normal distribution for the probability of *Hit* [i.e., signal present and the response is ‘present’], and *Z_FA_* is the same for *False Alarm* [i.e., signal absent but the response is ‘present’]) and *criterion* (*C* = -0.5 × (*Z_Hit_* + *Z_FA_*)). *Sensitivity d’* indicates the participant’s ability to discriminate signal from noise, and *criterion C* quantifies how liberal (i.e., *C* <0) or conservative (i.e., *C* >0) the response strategy (bias) may be. Both *d’* and *C* were normally distributed so no further transformations were performed.

Two language-related executive functions were assessed. Phonological memory (i.e., working memory in the auditory domain) was tested using the Non-Word Repetition task [77], consisting of 28 non-words. Participants were asked to listen carefully to a non-word (spoken in a British English accent) and repeat it immediately. Their utterance was audio-recorded and coded by a trained native British researcher using strict criteria: all vowels, consonants and accents in the repeated utterance needed to be exactly the same as the stimulus for the item to be coded as correct. Number of correct items was treated as the outcome measure. Second, the word generativity (F-A-S) task required the participant to produce as many words beginning with the letter ‘F’ as possible in one minute. Names, tense changes, plurals, derivatives and pronouns were not allowed. The same task was then performed with letters ‘A’ and ‘S’. Total words generated, excluding repetitions and those breaking rules, were treated as the outcome measure.

Motor executive function involving motor coordination, inhibition and planning was partially assessed by the ‘assembly’ subtask of the Purdue Pegboard Test [78]; see below for details.

#### Perceptual attention to detail

At the perceptual level, people with ASC have been reported to show a preference for, and superior attention to detail [79] on visuospatial tasks [80], [81]. This islet of superiority has been interpreted as reflecting weak central coherence (WCC) [82], [83], superior low-level processing in perceptual modalities (i.e., enhanced perceptual function) [84], [85], or superior discrimination (i.e., enhanced discrimination and reduced generalization) [86], [87].

Here we used the adult version of the Embedded Figures Test (EFT) [88] to investigate this domain of cognition. Similar to a previous study [81], we used ‘Form A’ which consisted of 12 figures in fixed order plus an additional practice item, each depicting a complex design and a simple shape which was hidden in the complex design. The participant was first shown and asked to study the complex design for no more than 15 seconds, then shown the simple shape (meanwhile the complex design was covered) for no more than 10 seconds. Timing (using a stopwatch) started when the complex design was shown again to the participant (meanwhile the simple shape was covered) and s/he was asked to identify the simple shape with a stylus pen. Time was noted (but not stopped) once the participant said s/he found the simple shape. If the answer was correct, the noted time was recorded. If the answer was incorrect then s/he was asked to find it again, and the final time was recorded when the identification was correct. Participants were given an upper limit of 120 seconds, and failure to find the simple shape within this allotted time was scored as a failure and the response time for the item was recorded as 120 seconds.

Two analysis strategies were employed, accounting for different aspects of the task performance [89]. First, average response time from all 12 items (including both correct and failure items) were used as the outcome measure, in order to account for both accuracy and response time; this has been commonly adopted in previous studies [81], [90]–[92]. Second, to purely assess performance speed on correct items, mean response time for correct items only were taken as the outcome measure; also in the following statistical modeling, accuracy was included as a covariate to reduce the influence of accuracy on response time, as suggested by White & Saldana [89].

#### Dexterity

Although not part of the current diagnostic criteria, motor clumsiness was regarded as a diagnostic feature by Hans Asperger in his seminal report on ‘autistic psychopathy’ [93]. Various motor anomalies (e.g. gross motor, fine motor, coordination, gait and posture, movement imitation) and dyspraxia have been associated with ASC [94]–[101]. Rigorous meta-analysis also suggests that motor coordination deficits are a pervasive feature of ASC [102]. However there is still a debate as to whether there is intrinsic atypical motor development or if the motor clumsiness reflects executive dysfunction.

Here we explored motor function using the Purdue Pegboard Test [78], a reliable and well-validated standard test for dexterity, involving both gross movement of arms, hands and fingers and fingertip dexterity. The test consists of four subtests: (i) ‘right-hand’: the participant is asked to insert small pins into holes on the board for 30 seconds using only their right hand, and the number of pins successfully placed in the holes is scored; (ii) ‘left-hand’: the same procedure is repeated using the left hand; (iii) ‘alternative/both-hands’: the same procedure is used but the participant is instructed to pick up and place pins in two rows of holes using both hands alternatively; and (iv) ‘assembly’: using both hands alternatively, the participant is asked to assemble sequences of pins, collars and washers for 60 seconds, and the number of all items successfully placed in the holes is recorded.

### Statistical Analysis

All analyses were performed under a two-way factorial analysis of variance framework, with sex and diagnosis as the two fixed factors, each with two levels. Due to potential interdependency among the outcome variables within each domain or outcome measures from the same task, multivariate analysis of covariance (MANCOVA) was first conducted for each domain or outcome measures from the same task, followed by individual ANCOVA if the overall MANCOVA yielded significant results. The alpha level for MANCOVA was adjusted using the Bonferroni method by the total number of MANCOVA performed (i.e., 0.05/5 = 0.01). The alpha level for the post-hoc ANCOVAs was corrected for multiple comparisons using the Bonferroni method within each MANCOVA (i.e., 0.05/number of ANCOVAs performed). For each ANCOVA, if significant, four planned comparisons were performed between (i) males with and without ASC, (ii) females with and without ASC, (iii) neurotypical males and females, and (iv) males and females with ASC; alpha level was set at 0.05/4 = 0.0125 by Bonferroni correction.

Age was positively correlated with most of the reaction time measures, and IQ positively correlated with most accuracy measures. Therefore for all analyses age and FIQ were included as covariates. As a supplement, we also performed all tests without covarying age and FIQ, and found that the group difference patterns and the statistical significance remained the same before and after including the covariates. Therefore, results from models covarying with age and FIQ are reported because they more accurately reflect standard practices in the literature [103], [104]. All statistical analyses were performed with the PASW Statistics version 18 (SPSS Inc., Chicago, IL, USA). A brief summary of descriptive statistics for raw scores in all cognitive tasks was provided in Table S1.

## Results

### Participant Characteristics

All groups (MC: neurotypical control male adults; MA: male adults with ASC; FC: neurotypical control female adults; FA: female adults with ASC) were matched on age and full-scale IQ. For subscales, FC scored higher than MC on verbal IQ and MC scored higher than FA on performance IQ under a non-corrected threshold of *p*<0.05. As reported earlier [15], for ADI-R MA scored marginally higher than FA in the RSB domain but they had the same severity on social reciprocity and communication domains. MA scored significantly higher than FA in ADOS scores even after correction for multiple comparisons. See Table 1.

**Table 1:** Demographic and behavioral characteristics.

	MC (N = 32)	MA (N = 32)	FC (N = 32)	FA (N = 32)	Statistics^2^
	Mean (SD)	Mean (SD) [range]^1^	Mean (SD)	Mean (SD) [range]^1^	
Age (Years)	28.7 (5.9)	27.0 (7.2)	27.6 (6.3)	28.1 (8.2)	ns
Verbal IQ	111.0 (12.2)	112.5 (14.4)	118.3 (10.1)	114.5 (15.4)	FC>MC (*p* = .030)
Performance IQ^3^	118.3 (11.5)	112.2 (15.3)	116.4 (9.4)	110.2 (17.0)	MC>FA (*p* = .019)
Full IQ^3^	116.3 (11.8)	113.7 (15.1)	119.7 (8.4)	114.1 (15.5)	ns
ADI-R^4^					
Social	–	18.0 (5.1) [10]–[27]	–	16.9 (4.8) [11]–[29]	ns
Communication	–	15.2 (3.5) [8]–[22]	–	13.6 (4.4) [8]–[25]	ns
RSB	–	5.8 (2.5) [2]–[10]	–	4.5 (2.0) [2]–[10]	MA>FA (*p* = .035)
ADOS^5^					
S + C	–	8.5 (4.8) [1]–[17]	–	4.6 (4.4) [0–19]	MA>FA (*p*<.001)
RSB	–	1.0 (1.0) [0–4]	–	0.1 (0.3) [0–1]	MA>FA (*p*<.001)

1 For ADI-R and ADOS scores.

2 Independent sample *t*-tests. All *p* values were **not** corrected for multiple comparisons.

3 Levene’s Test for Equality of Variances showed significant non-equal variances, therefore equal variance was not assumed in the statistical tests.

4 N = 32 for MA, N = 29 for FA.

5 Distribution of scores significantly deviant from normal, therefore non-parametric Mann-Whitney tests were performed for group comparison of ADOS algorithm scores.

MC = neurotypical control group male adults;

MA = male adults with ASC;

FC = neurotypical control group female adults;

FA = female adults with ASC;

SD = standard deviation; ns = non-significant (*p*>0.05);

ADI-R = Autism Diagnostic Interview-Revised; RSB: repetitive, restrictive and stereotyped behavior; ADOS: Autism Diagnostic Observation Schedule; S + C: ADOS ‘social interaction + communication’ total scores.

### Mentalizing and Emotion Perception

**Figure 1:**
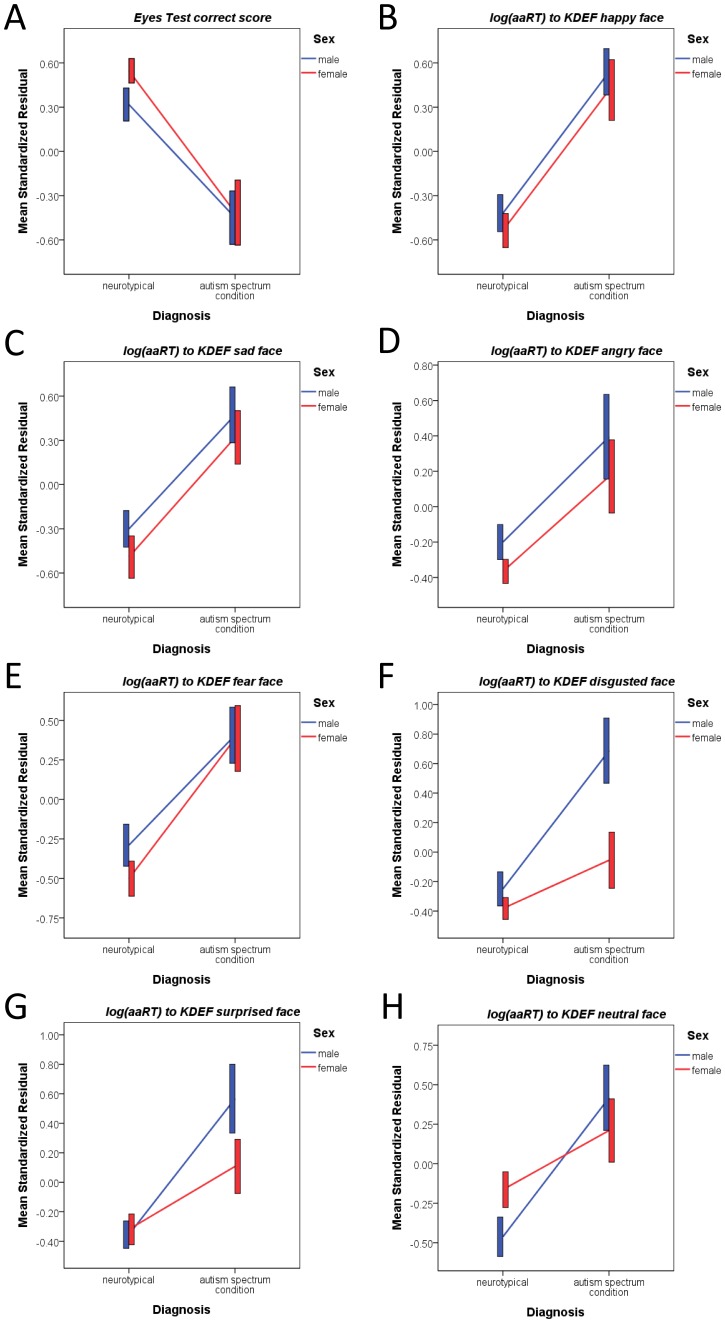
Eyes Test and KDEF Test performance. These line graphs show the performance on the Eyes Test and KDEF Test for the four groups. The graphs illustrate significant main effects of diagnosis across all outcome measures (A: Eyes Test correct score; B-H: log-transformed aaRT for KDEF Test emotion faces of happy, sad, angry, fear, disgusted, surprised and neutral faces) and a lack of a sex-by-diagnosis interaction. The y-axis plots the mean of standardized residual (i.e., after adjusted for all covariates in the model) ±1 standard error. The x-axis designates diagnostic group with the neurotypical control group on the left and ASC group on the right. Separate lines indicate males and female groups (males, blue; females, red).

Our first analysis was a MANCOVA with nine outcome measures from the Eyes Test and KDEF Test (Table 2) as dependent variables, sex and diagnosis as fixed factors, and age and FIQ as covariates. There was a significant main effect of diagnosis but not of sex, nor was there a significant interaction. Post-hoc univariate two-way factorial ANCOVAs showed that the main effect of diagnosis was evident for all measures except the log-transformed Eyes Test RT. There was no significant main effect of sex or sex-by-diagnosis interaction on any ANCOVA. See Table 2. This indicated that in general people with ASC were less accurate in advanced mentalizing and slower in identifying all basic facial emotions; see Figure 1. Post-hoc planned comparisons confirmed the presence of significant simple effects of diagnosis across both sexes for all variables, except non-significance in females for disgusted, surprised and neutral faces (panels F, G, H). Furthermore, there were no sex differences in the neurotypical group.

**Table 2:** MANCOVA and post-hoc ANCOVAs for the Eyes Test and the KDEF Test.

	Main effect of Diagnosis			Main effect of Sex			Interaction effect		
MANCOVA	*F*_(9,114)_	*p*	*V*	*F*_(9,114)_	*p*	*V*	*F*_(9,114)_	*p*	*V*
	7.773	<.001	.380	1.315	.237	.094	0.874	.551	.065
**ANCOVA**	***F*** **_(1,122)_**	***p***	***η_p_^2^***	***F*** **_(1,122)_**	***p***	***η_p_^2^***	***F*** **_(1,122)_**	***p***	***η_p_^2^***
Eyes Test									
Correct	30.30	<.001	.199	0.79	.375	.006	0.42	.521	.003
log(RT)	3.26	.074	.026	1.52	.221	.012	0.11	.745	.001
KDEF log(aaRT)									
Happy	38.95	<.001	.242	0.72	.398	.006	<0.01	.986	<.001
Sad	24.51	<.001	.167	1.25	.267	.010	0.02	.882	<.001
Angry	11.43	.001	.086	1.41	.238	.011	0.02	.881	<.001
Fear	24.41	<.001	.167	0.60	.439	.005	0.38	.537	.003
Disgusted	15.80	<.001	.115	7.53	.007	.058	3.45	.066	.028
Surprised	17.19	<.001	.123	1.76	.187	.014	2.18	.142	.018
Neutral	14.27	<.001	.105	0.06	.814	<.001	2.23	.138	.018

*V*: Pillai’s Trace *V*;

*η_p_^2^*: effect size partial eta-squared.

**Figure 2:**
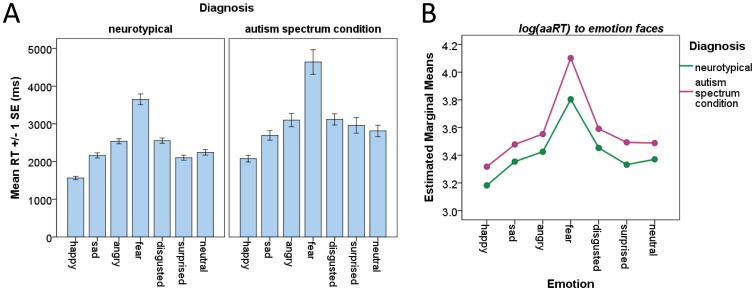
KDEF Test emotion-by-diagnosis interaction. Panel A shows the raw reaction times across all seven emotion faces for the neurotypical control group (left graph) and the ASC group (right graph). For both groups, fear (4th bars) required the longest time to identify, and happy (1st bars) the shortest. Error bars indicate (within-group) standard error of the mean. Panel B indicates that the ASC groups (purple line) required an even longer time than the control groups (green) to identify fear compared to all other emotion faces. There was no sex difference in this emotion-by-diagnosis interaction, so males and females are illustrated together.

To further explore if any particular emotion was specifically difficult for people with ASC, a mixed-model factorial ANCOVA was performed treating emotion as the within-subject factor (with seven levels), sex and diagnosis as the between-subject factors, and age and FIQ as covariates. Results showed a significant emotion-by-diagnosis interaction (*F*_(3.149, 384.133)_ = 3.854, *p* = 0.009; *df* was corrected using Greenhouse-Geisser estimates of sphericity due to a significant violation of the assumption of sphericity, Mauchly’s test *p*<0.001), but no emotion-by-sex or emotion-by-sex-by-diagnosis interactions. Contrasts revealed that people with ASC were particularly slower than controls in identifying fear compared to all other emotions: happy (*F*_(1,122)_ = 7.341, *p* = 0.008), sad (*F*_(1,122)_ = 8.468, *p* = 0.004), angry (*F*_(1,122)_ = 7.988, *p* = 0.006), disgusted (*F*_(1,122)_ = 7.638, *p* = 0.007), surprised (*F*_(1,122)_ = 3.609, *p* = 0.06), and neutral (*F*_(1,122)_ = 6.826, *p* = 0.01) faces; see Figure 2.

### Executive Function

**Figure 3:**
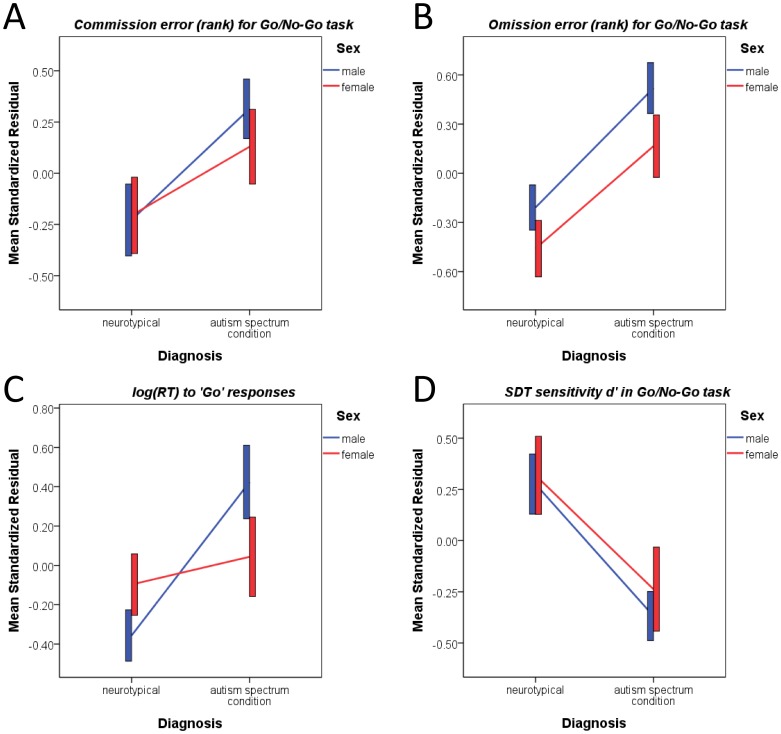
Go/No-Go task performance. The line graphs illustrate the main effect of diagnosis across outcome variables (A, B: rank-transformed commission and omission errors, the higher the more error; C: log-transformed RT for ‘go’ response; D: sensitivity *d’* derived from SDT). There were no sex-by-diagnosis interactions. Convention of the graphs is the same as that in Figure 1.

The Go/No-Go data for one male ASC participant was not recorded due to website failure, so there were only 31 male participants included in this analysis. We first performed an exploratory analysis on the conventional outcome measures of commission and omission errors. A MANCOVA treated the two rank-transformed error rates as dependent variables, sex and diagnosis as fixed factors, and age and FIQ as covariates. There was a significant main effect of diagnosis but not of sex, nor a significant interaction. Post-hoc univariate two-way factorial ANCOVAs showed that the main effect of diagnosis (i.e., people with ASC had more errors) was evident for both measures. There was no significant main effect of sex or sex-by-diagnosis interaction on either ANCOVA. See Table 3. Post-hoc planned comparisons showed that the simple effect of diagnosis was significant across sexes for omission error (in males *p* = 0.001, in females *p* = 0.010), but not for commission error in either sex (in males *p* = 0.022, in females *p* = 0.235). See Figure 3, panels A and B.

**Table 3:** MANCOVAs and post-hoc ANCOVAs for the executive function tasks.

	Main effect of Diagnosis			Main effect of Sex			Interaction effect		
MANCOVA	*F*_(2,120)_	*p*	*V*	*F*_(2,120)_	*p*	*V*	*F*_(2,120)_	*p*	*V*
Go/No-Go rank-transformederror rates	9.490	<.001	.137	1.749	.178	.028	0.173	.842	.003
**ANCOVA**	***F*** **_(1,121)_**	***p***	***η_p_^2^***	***F*** **_(1,121)_**	***p***	***η_p_^2^***	***F*** **_(1,121)_**	***p***	***η_p_^2^***
Commission	6.51	.012	.051	0.25	.621	.002	0.34	.564	.003
Omission	17.25	<.001	.125	3.52	.063	.028	0.08	.779	.001
**MANCOVA**	***F*** **_(3,119)_**	***p***	***V***	***F*** **_(3,119)_**	***p***	***V***	***F*** **_(3,119)_**	***p***	***V***
Go/No-Go re-analysis	7.688	<.001	.162	0.221	.881	.006	1.476	.225	.036
**ANCOVA**	***F*** **_(1,121)_**	***p***	***η_p_^2^***	***F*** **_(1,121)_**	***p***	***η_p_^2^***	***F*** **_(1,121)_**	***p***	***η_p_^2^***
log(RT) for ‘Go’ responses	7.33	.008	.057	0.14	.707	.001	3.41	.067	.027
SDT *d’*	12.75	.001	.095	0.31	.581	.003	0.06	.813	<.001
SDT *C*	0.84	.360	.007	0.16	.695	.001	0.28	.600	.002
**MANCOVA**	***F*** **_(2,121)_**	***p***	***V***	***F*** **_(2,121)_**	***p***	***V***	***F*** **_(2,121)_**	***p***	***V***
Language related tasks	0.681	.508	.011	4.337	.015	.067	0.767	.467	.013
**ANCOVA**	***F*** **_(1,122)_**	***p***	***η_p_^2^***	***F*** **_(1,122)_**	***p***	***η_p_^2^***	***F*** **_(1,122)_**	***p***	***η_p_^2^***
F-A-S	0.19	.668	.002	6.51	.012	.051	1.17	.282	.009
NWR	1.29	.258	.010	1.35	.248	.011	0.23	.635	.002

Data were re-analyzed with measures derived from SDT. A MANCOVA treated the log-transformed mean RT for all ‘go’ trials, sensitivity *d*’ and criterion *C* as dependent variables, sex and diagnosis as fixed factors, and age and FIQ as covariates. Echoing the exploratory analysis, there was a significant main effect of diagnosis but not of sex, nor was there a significant interaction. Post-hoc ANCOVAs showed that the main effect of diagnosis were only evident for log-transformed mean RT for ‘go’ responses and *d*’, but not for *C*. There was no significant main effect of sex or sex-by-diagnosis interaction on any ANCOVA. See Table 3. This indicated that in general people with ASC were slower in response to stimuli and less sensitive in discriminating signal from noise, yet their response style/bias was similar to controls. Post-hoc comparisons showed that the simple effect of diagnosis was significant in males but not in females for ‘go’ RT (in males *p* = 0.001, in females *p* = 0.458) and *d*’ (in males *p* = 0.001, in females *p* = 0.053); note that the sex difference of these diagnostic differences did not reach a significant sex-by-diagnosis interaction. See Figure 3, panels C and D.

Another MANCOVA was conducted for the language-related executive functions, treating Non-Word Repetition (NWR) and word generativity (F-A-S) task scores as the dependent variables, sex and diagnosis the fixed factors, and age and FIQ the covariates. There was a marginal significant main effect of sex but not of diagnosis, nor was there a significant interaction. Post-hoc ANCOVAs showed that the main effect of sex was only evident for F-A-S (males better than females) but not for NWR scores, and confirmed no main effect of diagnosis or interaction effect in either of them. See Table 3.

### Perceptual Attention to Detail

**Figure 4:**
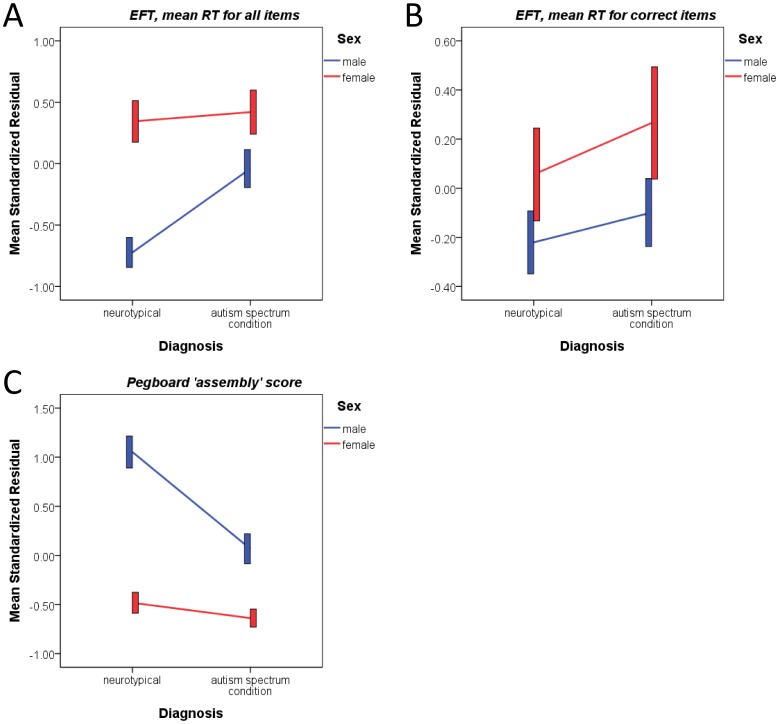
EFT and Purdue Pegboard Test assembly performances. Both EFT RT for all items (reflecting mainly accuracy, panel A) and assembly subtest score in the Purdue Pegboard Test (panel C) showed a significant interaction between sex and diagnosis. Males with ASC on average performed worse than neurotypical males, but females with ASC performed equally well as neurotypical females. EFT RT for correct items (reflecting purely processing speed, panel B) showed only a main effect of sex that overall females were on average slower than males. Convention of the graphs is the same as that in Figure 1.

An ANCOVA was performed for the *mean RT for all items* in EFT, with sex and diagnosis as fixed factors, and age and FIQ as covariates. We noted significant main effects of sex and diagnosis, and a marginal interaction. FIQ explained most of the variance in the model (*F*_(1,122)_ = 137.400, *p*<0.001, *η_p_^2^* = 0.530). Planned comparisons indicated that adult males with ASC performed worse than typical males (*p* = 0.001), which was not the case between the female groups (*p* = 0.828). Sex differences were observed in the control groups (*p*<0.001) but not in the ASC groups (*p* = 0.046). See Table 4 and Figure 4, panel A. Similar to what was found in a previous study [89], this all-item mean RT was highly correlated with accuracy (Spearman’s *ρ* = -0.93, *p*<0.001) so should be viewed as mainly reflecting information about accuracy, which itself was distributed far from normality and could not be analyzed adequately within the current framework.

**Table 4:** ANCOVAs for EFT scores.

	Main effect of Diagnosis			Main effect of Sex			Interaction effect		
ANCOVA	*F*_(1,122)_	*p*	*η_p_^2^*	*F*_(1,122)_	*p*	*η_p_^2^*	*F*_(1,122)_	*p*	*η_p_^2^*
Mean RT for all items	5.54	.020	.043	22.94	<.001	.158	3.70	.057	.029
**ANCOVA**	***F*** **_(1,118)_**	***p***	***η_p_^2^***	***F*** **_(1,118)_**	***p***	***η_p_^2^***	***F*** **_(1,118)_**	***p***	***η_p_^2^***
Mean RT for correct items	1.15	.286	.010	4.06	.046	.033	0.01	.927	<.001

To explore performance speed, a second ANCOVA was conducted for the *mean RT for correct items* only, with the same fixed factors and covariates but included accuracy (log-transformation of the reflected accuracy, i.e., subtracted from 2, to approximate normality) as an additional covariate to reduce the influence of different accuracy on performance speed [89]. Two males and one female with ASC were excluded because they failed all 12 items. We found a significant main effect of sex but not for diagnosis; there was no significant interaction. FIQ still explained most of the variance in the model (*F*_(1,118)_ = 16.992, *p*<0.001, *η_p_^2^* = 0.126). Post-hoc comparisons showed non-significant sex differences in both typical control (*p* = 0.181) and ASC groups (*p* = 0.171). See Table 4 and Figure 4, panel B.

### Motor Function

A MANCOVA treated the four outcome measures of the Purdue Pegboard Test (i.e., ‘right-hand’, ‘left-hand’, ‘alternative/both-hands’ and ‘assembly’ scores) as dependent variables, sex and diagnosis as fixed factors, and age and FIQ as covariates. There were significant main effects of sex, diagnosis, and a sex-by-diagnosis interaction. Post-hoc ANCOVAs indicated that the significance was driven by the assembly score with significant effects of sex, diagnosis, and an interaction; none of these were significant for the other three measures. See Table 5. Planned comparisons indicated that for the assembly subtest, males with ASC performed worse than typical males (*p*<0.001), which was not the case between the female groups (*p* = 0.200). A sex difference was observed in both the control (*p*<0.001) and ASC groups (*p*<0.001). See Figure 4, panel C.

**Table 5:** MANCOVA and post-hoc ANCOVAs for Purdue Pegboard Test scores.

	Main effect of Diagnosis			Main effect of Sex			Interaction effect		
MANCOVA	*F*_(4,119)_	*p*	*V*	*F*_(4,119)_	*p*	*V*	*F*_(4,119)_	*p*	*V*
	8.280	<.001	.218	25.229	<.001	.459	6.178	<.001	.172
**ANCOVA**	***F*** **_(1,122)_**	***p***	***η_p_^2^***	***F*** **_(1,122)_**	***p***	***η_p_^2^***	***F*** **_(1,122)_**	***p***	***η_p_^2^***
Right-hand	6.31	.013	.049	0.24	.628	.002	0.09	.770	.001
Left-hand	5.03	.027	.040	0.94	.333	.008	0.02	.902	<.001
Both-hands	0.28	.599	.002	5.24	.024	.041	1.79	.184	.014
Assembly	18.02	<.001	.129	70.82	<.001	.367	9.90	.002	.075

We further repeated the ANCOVA on the assembly score by additionally entering either the right-hand or left-hand subtest scores as a covariate to control for basic motor-speed difference. These did not change the pattern of group differences and statistical significance.

## Discussion

Previously, we demonstrated behavioral sex differences between males and females with ASC, all of whom were high-functioning adults, showing that females displayed fewer autistic behaviors during interpersonal interaction, but nevertheless reported more autistic traits and sensory issues [15]. The current study further demonstrates that ASC varies with sex in some non-social cognitive domains, although not in relation to the core social cognitive difficulties.

### Sex-by-diagnosis Interactions are Domain-dependent

#### Mentalizing and emotion perception

Regardless of sex, adults with ASC showed impaired mentalizing and basic facial emotion recognition abilities relative to neurotypical adults. This is not surprising given this cognitive domain critically underlies the socio-communication difficulties by which ASC is defined [40], [105]. In contrast, we found a striking sex difference in current interactive behaviors on the ADOS, with milder interpersonal autistic features in women with ASC compared to men with ASC [15]. These results strengthen the arguments for superficial camouflaging of social-communication difficulties in females with ASC [2], [4], [15], [106].

The deficit in advanced mentalizing in adults with ASC replicates previous studies [39], [55], [62], [107]. For basic emotion recognition, they also showed difficulty in recognizing emotion faces across *all* basic emotions. This is in contrast with previous studies that either found impairments on negative but not positive emotions [56]–[61], or reported comparable behavioral performance on basic facial emotion recognition in adolescents and adults with ASC [108]–[111]. At least two other reasons could potentially contribute to this observed difficulty, beyond basic emotion recognition ability *per se*: a generally slower stimulus-reaction response, and/or difficulties in processing faces. We did not have a measure of simple reaction time on a neutral stimulus-response task. However, we did measure the ‘go’ RT in the Go/No-Go task, which indicated stimulus-reaction response in a simple non-social task. Here we found that people with ASC performed slower, and this ‘go’ RT was moderately correlated with all KDEF Test RTs. As an additional test, we corrected each emotion RT according to the ‘go’ RT for individual participants (i.e., dividing each emotion RT by the individual’s ‘go’ RT), and the pattern of group differences remained the same, with a significant main effect of diagnosis across all emotions (happy: *p*<0.001, sad: *p* = 0.001, angry: *p* = 0.014, fear: *p*<0.001, disgusted: *p* = 0.004, surprised: *p* = 0.001, neutral: *p* = 0.009). This additional test suggests that, even after controlling for generally slower stimulus-reaction response, adults with ASC are still slower in recognizing all basic emotion faces relative to neurotypical adults. Thus, rather than inferring that individuals with ASC are generally slower irrespective of task, the fact that these deficits remain even after accounting for general ‘slowness’ argues for some specificity in deficits within the KDEF test.

To what extent this impairment may be accounted for by difficulties in face processing *per se* is unclear. Such an influence may exist given earlier reports of face processing difficulties in people with ASC [112]–[120]. However, a recent report also shows independent facial identity vs. facial emotion processing in children with ASC [121]. Whether atypical facial emotion processing in ASC is primary or secondary to other social deficits in ASC (e.g. face processing difficulties, low interest/motivation in social interaction, and/or reduced scanning of other’s eyes) requires more investigation [122].

We also found that adults with ASC (both males and females) were particularly impaired in identifying fear, compared to other emotion faces. This is partially in line with previous reports on the difficulty in processing fear for people with ASC [56]–[59], and may implicate atypical amygdala function [123]–[125]. Another explanation for specific difficulties with fear may be due to a lack of attention to the eye region of the face. Prior work by Adolphs and colleagues [125] showed that bilateral amygdala lesions prevent recognition of fear in faces due to a lack of attention to the eyes. When attention is re-focused to the eyes, fear recognition returns to normal levels. Thus, given individuals with ASC tend to look less at the eyes [126], the deficit observed here for fear may reflect a lack of attention to the most salient feature of a fear face (the eyes).

#### Executive function

Within the domain of executive function we examined simple response inhibition, phonological memory, generativity and motor coordination/sequencing. Phonological memory and (word) generativity were not impaired in ASC. However, we found reduced sensitivity to signal detection (which is also reflected in more omission errors) in both sexes with ASC (the impairment being slightly smaller in females). We also found a slight impairment in simple response inhibition (reflected in commission errors) in ASC. In addition, on the dexterity subtest of assembly (which requires motor coordination, inhibition and planning on top of basic motor speed) there was a significant sex-by-diagnosis interaction: males with ASC were impaired relative to typical males, whilst females with or without ASC performed equally well. This difference is hard to account for purely in terms of gross or fine motor deficits, since a diagnostic group difference was not observed on any of the other simple dexterity subtests. Additionally, after controlling for basic motor speed, the group difference pattern on the assembly subtest remained the same.

In sum, we replicated earlier reports that, in ASC, phonological working memory and word generativity are intact [37], [73], [75], but found deficits in both sexes within ASC in simple response inhibition and in sensitivity to signals (less evident in females). Most interestingly, we found a strong sex-by-diagnosis interaction on the dexterity subtest involving motor executive function. This has implications that should be tested in the future: this might predict greater levels of dyspraxia in males with ASC, relative to females. Indeed, in a large sample of children and adolescents, females with ASC have been found to have less fine motor impairment [23].

Sex differences within ASC in executive function have been reported in children and adolescents [33]–[36]. Although performance across ages is difficult to compare due to developmental change, the message from the present and earlier reports is that sex differences within ASC exist in certain aspects of executive functions. The lower sensitivity to signal detection, alongside comparable response strategies in ASC, is of additional interest. This observation is solely based on the Go/No-Go task. Whether this lower signal sensitivity extends to other sub-domains of executive function or other cognitive domains will be important to establish.

Finally, our participants with ASC performed similarly to neurotypical adults on two language-related executive functions: phonological working memory and word generativity. The former has been reported to be impaired in adolescents with ASC with concurrent language impairment [127], but the latter is inconsistent in ASC [73], [128]. Our null finding is most likely to be attributable to sample characteristics - they were all high-functioning adults *without* current language impairments, so executive functions subserving language processing were likely to be unimpaired. This again reflects the considerable heterogeneity within ASC.

#### Perceptual attention to detail

Previous reports of EFT performance in ASC are inconsistent [89]. This may be due to different methodological and analytical strategies, as well as heterogeneity in central coherence within ASC [89]. On both outcome measures, i.e., mean RT for all items (reflecting mainly accuracy) and mean RT for correct items adjusted by accuracy (reflecting mainly processing speed), we replicated the typical male advantage [129]. Furthermore in the ASC group, the trend level significance (*p*<0.046) of male advantage on mean RT for all items corresponds to previous reports of better performance in males than females with ASC on the Block Design task in children [34] and adolescents [35]. On the other hand, we did not replicate the ASC superiority (over neurotypical controls) noted by some early studies [80], [81]. The results instead are in line with a well-powered study in children showing no such superiority on EFT performance [89].

Regarding RT for all items, males with ASC performed worse than typical males, but females with and without ASC performed comparably. This sex difference within ASC in EFT accuracy suggests that performance in visuospatial attention to detail may characterize men with ASC, but not women with ASC. The lack of superiority (or even worse performance) in ASC also challenges a prediction from the WCC theory. However, whether EFT really disentangles global from local processing is unclear, so interpretations should be made cautiously [89].

#### Motor function

In contrast to previous findings [102] we failed to find diagnostic group differences in general motor clumsiness on simple dexterity subtests (i.e., right-hand, left-hand and alternative/both-hands scores on the Purdue Pegboard Test). This is likely to be due to the fact that we recruited high-functioning adults and hardly any of them had a history of motor delay. Neurological comorbidities, including motor clumsiness, are usually associated with the more severe behavioral phenotype in ASC and lower IQ [99], [130]. Hence it is plausible that these high-functioning adults do not have basic motor impairments.

However, we did find a sex-by-diagnosis interaction on the assembly subtest, which involves a certain degree of motor coordination, planning and inhibition, in addition to basic motor skills. Poor performance in assembly but not other simpler subtests may thus reflect motor executive dysfunction. This was only found in males, but not females, with ASC. This warrants a detailed investigation of how the sex-specificity of motor executive dysfunction arises.

We did not find a female advantage on the assembly subtest in typical controls, in contrast to some previous reports [131], even when controlling for basic motor speed (reflected by right-hand or left-hand scores). This may be due to different sample characteristics (e.g., no laborer volunteers participated in the present study).

### Cross-domain Summary

Each cognitive domain was investigated by a small number of tasks, hence a cross-domain summary must be preliminary. The results nevertheless provide initial evidence that both cognitive similarities and differences between the sexes exist in adults with ASC and with average or above-average IQ. Males and females who have comparable childhood autistic symptoms currently show similar cognitive deficits in social domains, but are different in certain non-social domains.

Regarding difficulties in social cognition, the hallmark of ASC, males and females share the same level of deficit. This may be interpreted within the context that males and females with ASC are currently diagnosed using the same behavioral criteria, and one major component of it (social-communication difficulties) is closely tied to social cognition. On the other hand, in cognitive domains not directly related to diagnosing ASC, the pattern appears different. For visuospatial attention to detail and certain aspects of executive function, when there is a diagnostic group difference in performance, it is usually accompanied by a disordinal sex-by-diagnosis interaction: the effect of the diagnosis is seen in males but not in females. These interactions mostly occur when there is a sex difference (males outperforming females) in the neurotypical groups, implying that ASC may have more detrimental effects on males on domains in which they typically show superiority.

This is in contrast to the long-held view, mainly derived from observations of lower-functioning children, that females with ASC are more cognitively impaired than their male counterparts [19], [132]–[135]. According to this view, the present study should have shown greater impairment in females than in males. One reason for the new observation here that males with ASC are more impaired may be due to the fact that we studied high-functioning adults. In our view, the long-held view of females being more severely affected should not be taken as definite because the sex-differential influences of ASC may be substantially affected by other factors, particularly intellectual ability and age. This heterogeneity regarding how sex and ASC diagnosis interact warrants large-sample studies that include a wider range of IQ and age and equal-sized males and females.

In sum, we observed that for high-functioning adults, the expression of ASC is modulated by sex particularly for non-social cognition, which echoes recent reports showing sex differences within ASC in executive function and visuospatial processing in children and adolescents [33]–[36]. These results have implications both for informing our understanding of mechanisms, and for clinical practice. Mechanistically, it sheds light on how ASC develops and manifests differentially by sex. Clinically, it suggests that sex-specific cognitive assessments may be useful. For instance, social-cognitive tasks may be helpful in identifying ASC-related difficulties for both sexes. However, when interpreting results from executive function and visuospatial processing tasks, sex-specificity should be taken into account. Our findings also suggest that higher-level motor function should be regularly assessed for people with high-functioning ASC. The other clinical implication is that we may need both sex-general and sex-specific intervention strategies. For example, social-cognitive enhancement may be helpful for both sexes, yet for males remediation for the non-social domains may be particularly important.

### Limitations and Future Directions

The findings have several limitations. First, we only tested adults with ASC who did not have intellectual disability or other common comorbidities (e.g. epilepsy, hyperkinetic disorder). Whether (and how) these co-occurring conditions may further modulate cognition in ASC differentially for males and females is unknown. Therefore the findings may not generalize to other subgroups with ASC. Secondly, not all cognitive domains key to ASC in adults were examined in detail. For example, we did not assess advanced language function, particularly semantics and pragmatics, which are likely to be atypical in high-functioning adults with ASC; certain sub-domains of executive function commonly reported to be impaired in ASC (i.e., set-shifting and planning) were also not specifically examined. A Navon task [136] may have been more sensitive to global-local attention bias than the EFT, and the spontaneous ToM [54] and moral judgment ToM tasks [53] inform different aspects of mentalizing in high-functioning adults. Thirdly, to fully address the relationship between sex and ASC it is best to have tasks showing the largest effect sizes for typical sex differences, such as those measuring visuospatial abilities of targeting, mental rotation, and aggression [129], [137]. Future larger-scale research should utilize a more comprehensive battery to pinpoint the fine-grained sex differences in various cognitive domains. Fourthly, the distribution of age varies substantially in our study. Although the influence of age on cognitive performances should have been well controlled by age-matching across groups and by including age as a covariate, we did not examine potential age-by-sex, age-by-diagnosis, or age-by-sex-by-diagnosis interactions. These types of nested interactions between age and the factors of interest would require investigation in much larger cohorts. Lastly, due to the limited sample size per group, there is the potential that we had limited power to detect small effect sizes in the sex-by-diagnosis interactions and these smaller effects may be deemed important in further studies with increases in sample size. Therefore, the null findings should be interpreted with caution and should not be considered unequivocal proof of a lack of sex difference regarding how cognitive characteristics are affected by ASC.

## Supporting Information

Table S1 A brief summary of the descriptive statistics for raw scores in all the cognitive tasks.
(DOC)

## References

[1] Fombonne E (2005) Epidemiological studies of pervasive developmental disorder. In: Volkmar F, Paul R, Klin A, Cohen D, editors. Handbook of Autism and Pervasive Developmental Disorders. Hoboken, NJ: Wiley. 42–69.

[2] Attwood T (2006) The pattern of abilities and development of girls with Asperger’s syndrome. Asperger’s and girls. Arlington, TX: Future Horisons, Inc.

[3] Kopp S, Gillberg C (1992) Girls with social deficits and learning problems: Autism, atypical Asperger syndrome or a variant of these conditions. European Child & Adolescent Psychiatry 1: 89–99. 10.1007/BF02091791 29871391

[4] Baron-Cohen S, Lombardo MV, Auyeung B, Ashwin E, Chakrabarti B, et al (2011) Why are autism spectrum conditions more prevalent in males? PLoS Biol 9: e1001081. 21695109 10.1371/journal.pbio.1001081 PMC3114757

[5] Kopp S, Gillberg C (2011) The Autism Spectrum Screening Questionnaire (ASSQ)-Revised Extended Version (ASSQ-REV): An instrument for better capturing the autism phenotype in girls? A preliminary study involving 191 clinical cases and community controls. Res Dev Disabil 32: 2875–2888. 21664105 10.1016/j.ridd.2011.05.017

[6] Skuse DH (2007) Rethinking the nature of genetic vulnerability to autistic spectrum disorders. Trends Genet 23: 387–395. 17630015 10.1016/j.tig.2007.06.003

[7] Constantino JN (2011) The quantitative nature of autistic social impairment. Pediatr Res 69: 55R–62R. 10.1203/PDR.0b013e318212ec6e PMC3086844 21289537

[8] Wolff S, McGuire RJ (1995) Schizoid personality in girls: a follow-up study–what are the links with Asperger’s syndrome? J Child Psychol Psychiatry 36: 793–817. 7559846 10.1111/j.1469-7610.1995.tb01330.x

[9] Dworzynski K, Ronald A, Bolton P, Happe F (2012) How different are girls and boys above and below the diagnostic threshold for autism spectrum disorders? J Am Acad Child Adolesc Psychiatry 51: 788–797. 22840550 10.1016/j.jaac.2012.05.018

[10] Mattila ML, Kielinen M, Linna SL, Jussila K, Ebeling H, et al.. (2011) Autism spectrum disorders according to DSM-IV-TR and comparison with DSM-5 draft criteria: An epidemiological study. J Am Acad Child Adolesc Psychiatry 50: 583–592 e511. 10.1016/j.jaac.2011.04.001 21621142

[11] Kim YS, Leventhal BL, Koh YJ, Fombonne E, Laska E, et al (2011) Prevalence of autism spectrum disorders in a total population sample. Am J Psychiatry 168: 904–912. 21558103 10.1176/appi.ajp.2011.10101532

[12] Idring S, Rai D, Dal H, Dalman C, Sturm H, et al (2012) Autism spectrum disorders in the stockholm youth cohort: design, prevalence and validity. PLoS ONE 7: e41280. 22911770 10.1371/journal.pone.0041280 PMC3401114

[13] Zwaigenbaum L, Bryson SE, Szatmari P, Brian J, Smith IM, et al.. (2012) Sex Differences in Children with Autism Spectrum Disorder Identified Within a High-Risk Infant Cohort. J Autism Dev Disord. doi: 10.1007/s10803-012-1515-y. 10.1007/s10803-012-1515-y 22453928

[14] Wing L, Gould J, Gillberg C (2011) Autism spectrum disorders in the DSM-V: better or worse than the DSM-IV? Res Dev Disabil 32: 768–773. 21208775 10.1016/j.ridd.2010.11.003

[15] Lai MC, Lombardo MV, Pasco G, Ruigrok AN, Wheelwright SJ, et al (2011) A behavioral comparison of male and female adults with high functioning autism spectrum conditions. PLoS ONE 6: e20835. 21695147 10.1371/journal.pone.0020835 PMC3113855

[16] Holtmann M, Bolte S, Poustka F (2007) Autism spectrum disorders: sex differences in autistic behaviour domains and coexisting psychopathology. Dev Med Child Neurol 49: 361–366. 17489810 10.1111/j.1469-8749.2007.00361.x

[17] Pilowsky T, Yirmiya N, Shulman C, Dover R (1998) The Autism Diagnostic Interview-Revised and the Childhood Autism Rating Scale: differences between diagnostic systems and comparison between genders. J Autism Dev Disord 28: 143–151. 9586776 10.1023/a:1026092632466

[18] Solomon M, Miller M, Taylor SL, Hinshaw SP, Carter CS (2012) Autism symptoms and internalizing psychopathology in girls and boys with autism spectrum disorders. J Autism Dev Disord 42: 48–59. 21442362 10.1007/s10803-011-1215-z PMC3244604

[19] Tsai LY, Beisler JM (1983) The development of sex differences in infantile autism. Br J Psychiatry 142: 373–378. 6850175 10.1192/bjp.142.4.373

[20] Carter AS, Black DO, Tewani S, Connolly CE, Kadlec MB, et al (2007) Sex differences in toddlers with autism spectrum disorders. J Autism Dev Disord 37: 86–97. 17216333 10.1007/s10803-006-0331-7

[21] Hartley SL, Sikora DM (2009) Sex differences in autism spectrum disorder: an examination of developmental functioning, autistic symptoms, and coexisting behavior problems in toddlers. J Autism Dev Disord 39: 1715–1722. 19582563 10.1007/s10803-009-0810-8 PMC3590797

[22] McLennan JD, Lord C, Schopler E (1993) Sex differences in higher functioning people with autism. J Autism Dev Disord 23: 217–227. 8331044 10.1007/BF01046216

[23] Mandy W, Chilvers R, Chowdhury U, Salter G, Seigal A, et al (2012) Sex differences in autism spectrum disorder: evidence from a large sample of children and adolescents. J Autism Dev Disord 42: 1304–1313. 21947663 10.1007/s10803-011-1356-0

[24] Schwarz E, Guest PC, Rahmoune H, Wang L, Levin Y, et al (2011) Sex-specific serum biomarker patterns in adults with Asperger’s syndrome. Mol Psychiatry 16: 1213–1220. 20877284 10.1038/mp.2010.102

[25] Gilman SR, Iossifov I, Levy D, Ronemus M, Wigler M, et al (2011) Rare de novo variants associated with autism implicate a large functional network of genes involved in formation and function of synapses. Neuron 70: 898–907. 21658583 10.1016/j.neuron.2011.05.021 PMC3607702

[26] Szatmari P, Liu XQ, Goldberg J, Zwaigenbaum L, Paterson AD, et al (2012) Sex differences in repetitive stereotyped behaviors in autism: Implications for genetic liability. Am J Med Genet B Neuropsychiatr Genet 159B: 5–12. 22095612 10.1002/ajmg.b.31238

[27] Puleo CM, Schmeidler J, Reichenberg A, Kolevzon A, Soorya LV, et al (2012) Advancing paternal age and simplex autism. Autism 16: 367–380. 22180389 10.1177/1362361311427154

[28] Beacher FD, Minati L, Baron-Cohen S, Lombardo MV, Lai MC, et al (2012) Autism attenuates sex differences in brain structure: a combined voxel-based morphometry and diffusion tensor imaging study. AJNR Am J Neuroradiol 33: 83–89. 22173769 10.3174/ajnr.A2880 PMC6345364

[29] Bloss CS, Courchesne E (2007) MRI neuroanatomy in young girls with autism: a preliminary study. J Am Acad Child Adolesc Psychiatry 46: 515–523. 17420687 10.1097/chi.0b013e318030e28b

[30] Schumann CM, Barnes CC, Lord C, Courchesne E (2009) Amygdala enlargement in toddlers with autism related to severity of social and communication impairments. Biol Psychiatry 66: 942–949. 19726029 10.1016/j.biopsych.2009.07.007 PMC2795360

[31] Schumann CM, Bloss CS, Barnes CC, Wideman GM, Carper RA, et al (2010) Longitudinal magnetic resonance imaging study of cortical development through early childhood in autism. J Neurosci 30: 4419–4427. 20335478 10.1523/JNEUROSCI.5714-09.2010 PMC2859218

[32] Nordahl CW, Lange N, Li DD, Barnett LA, Lee A, et al (2011) Brain enlargement is associated with regression in preschool-age boys with autism spectrum disorders. Proc Natl Acad Sci U S A 108: 20195–20200. 22123952 10.1073/pnas.1107560108 PMC3250128

[33] Nyden A, Hjelmquist E, Gillberg C (2000) Autism spectrum and attention-deficit disorders in girls. Some neuropsychological aspects. Eur Child Adolesc Psychiatry 9: 180–185. 11095040 10.1007/s007870070041

[34] Koyama T, Kamio Y, Inada N, Kurita H (2009) Sex differences in WISC-III profiles of children with high-functioning pervasive developmental disorders. J Autism Dev Disord 39: 135–141. 18629624 10.1007/s10803-008-0610-6

[35] Bolte S, Duketis E, Poustka F, Holtmann M (2011) Sex differences in cognitive domains and their clinical correlates in higher-functioning autism spectrum disorders. Autism 15: 497–511. 21454389 10.1177/1362361310391116

[36] Lemon JM, Gargaro B, Enticott PG, Rinehart NJ (2011) Executive functioning in autism spectrum disorders: a gender comparison of response inhibition. J Autism Dev Disord 41: 352–356. 20506000 10.1007/s10803-010-1039-2

[37] Boucher J (2009) The autistic spectrum: Characteristics, causes and practical issues. London, UK: SAGE Publications Ltd.

[38] Charman T, Jones CR, Pickles A, Simonoff E, Baird G, et al (2011) Defining the cognitive phenotype of autism. Brain Res 1380: 10–21. 21029728 10.1016/j.brainres.2010.10.075

[39] Losh M, Adolphs R, Poe MD, Couture S, Penn D, et al (2009) Neuropsychological profile of autism and the broad autism phenotype. Arch Gen Psychiatry 66: 518–526. 19414711 10.1001/archgenpsychiatry.2009.34 PMC2699548

[40] American Psychiatric Association (2000) Diagnostic and Statistical Manual of Mental Disorders, 4th edition text revision (DSM-IV-TR). Washington, DC: American Psychiatric Publishing, Inc.

[41] World Health Organization (1992) The ICD-10 classification of mental and behavioural disorders: Clinical descriptions and diagnostic guidelines. Geneva: World Health Organization.

[42] Lord C, Rutter M, Le Couteur A (1994) Autism Diagnostic Interview-Revised: a revised version of a diagnostic interview for caregivers of individuals with possible pervasive developmental disorders. J Autism Dev Disord 24: 659–685. 7814313 10.1007/BF02172145

[43] Ecker C, Suckling J, Deoni S, Lombardo MV, Bullmore E, et al (2012) Brain anatomy and its relationship to behavior in adults with autism spectrum disorder: A multi-center magnetic resonance imaging study. Arch Gen Psychiatry 69: 195–209. 22310506 10.1001/archgenpsychiatry.2011.1251

[44] Lai MC, Lombardo MV, Chakrabarti B, Sadek SA, Pasco G, et al (2010) A shift to randomness of brain oscillations in people with autism. Biol Psychiatry 68: 1092–1099. 20728872 10.1016/j.biopsych.2010.06.027

[45] Lombardo MV, Chakrabarti B, Bullmore ET, Baron-Cohen S (2011) Specialization of right temporo-parietal junction for mentalizing and its relation to social impairments in autism. Neuroimage 56: 1832–1838. 21356316 10.1016/j.neuroimage.2011.02.067

[46] Lombardo MV, Chakrabarti B, Bullmore ET, Sadek SA, Pasco G, et al (2010) Atypical neural self-representation in autism. Brain 133: 611–624. 20008375 10.1093/brain/awp306

[47] Lord C, Risi S, Lambrecht L, Cook EH Jr, Leventhal BL, et al (2000) The autism diagnostic observation schedule-generic: a standard measure of social and communication deficits associated with the spectrum of autism. J Autism Dev Disord 30: 205–223. 11055457

[48] Baron-Cohen S, Wheelwright S, Robinson J, Woodbury-Smith M (2005) The Adult Asperger Assessment (AAA): a diagnostic method. J Autism Dev Disord 35: 807–819. 16331530 10.1007/s10803-005-0026-5

[49] Boucher J (2012) Putting theory of mind in its place: psychological explanations of the socio-emotional-communicative impairments in autistic spectrum disorder. Autism 16: 226–246. 22297199 10.1177/1362361311430403

[50] Tantam D (2009) Can the world afford autistic spectrum disorder? London, UK: Jessica Kingsley Publishers.

[51] Trevarthen C, Aitken KJ (2001) Infant intersubjectivity: research, theory, and clinical applications. J Child Psychol Psychiatry 42: 3–48. 11205623

[52] Baron-Cohen S, Leslie AM, Frith U (1985) Does the autistic child have a “theory of mind”? Cognition 21: 37–46. 2934210 10.1016/0010-0277(85)90022-8

[53] Moran JM, Young LL, Saxe R, Lee SM, O’Young D, et al (2011) Impaired theory of mind for moral judgment in high-functioning autism. Proc Natl Acad Sci U S A 108: 2688–2692. 21282628 10.1073/pnas.1011734108 PMC3041087

[54] Senju A (2011) Spontaneous theory of mind and its absence in autism spectrum disorders. Neuroscientist 18: 108–113. 21609942 10.1177/1073858410397208 PMC3796729

[55] Baron-Cohen S, Wheelwright S, Hill J, Raste Y, Plumb I (2001) The “Reading the Mind in the Eyes” Test revised version: a study with normal adults, and adults with Asperger syndrome or high-functioning autism. J Child Psychol Psychiatry 42: 241–251. 11280420

[56] Ashwin C, Chapman E, Colle L, Baron-Cohen S (2006) Impaired recognition of negative basic emotions in autism: a test of the amygdala theory. Soc Neurosci 1: 349–363. 18633799 10.1080/17470910601040772

[57] Corden B, Chilvers R, Skuse D (2008) Avoidance of emotionally arousing stimuli predicts social-perceptual impairment in Asperger’s syndrome. Neuropsychologia 46: 137–147. 17920642 10.1016/j.neuropsychologia.2007.08.005

[58] Howard MA, Cowell PE, Boucher J, Broks P, Mayes A, et al (2000) Convergent neuroanatomical and behavioural evidence of an amygdala hypothesis of autism. Neuroreport 11: 2931–2935. 11006968 10.1097/00001756-200009110-00020

[59] Humphreys K, Minshew N, Leonard GL, Behrmann M (2007) A fine-grained analysis of facial expression processing in high-functioning adults with autism. Neuropsychologia 45: 685–695. 17010395 10.1016/j.neuropsychologia.2006.08.003

[60] Wallace GL, Case LK, Harms MB, Silvers JA, Kenworthy L, et al (2011) Diminished sensitivity to sad facial expressions in high functioning autism spectrum disorders is associated with symptomatology and adaptive functioning. J Autism Dev Disord 41: 1475–1486. 21347615 10.1007/s10803-010-1170-0 PMC3448486

[61] Law Smith MJ, Montagne B, Perrett DI, Gill M, Gallagher L (2010) Detecting subtle facial emotion recognition deficits in high-functioning Autism using dynamic stimuli of varying intensities. Neuropsychologia 48: 2777–2781. 20227430 10.1016/j.neuropsychologia.2010.03.008

[62] Golan O, Baron-Cohen S (2006) Systemizing empathy: teaching adults with Asperger syndrome or high-functioning autism to recognize complex emotions using interactive multimedia. Dev Psychopathol 18: 591–617. 16600069 10.1017/S0954579406060305

[63] Lundqvist D, Flykt A, Ohman A (1998) The Karolinska Directed Emotional Faces - KDEF. Stockholm, Sweden: Psychology Section, Department of Clinical Neuroscience, Karolinska Institute.

[64] Sutherland A, Crewther DP (2010) Magnocellular visual evoked potential delay with high autism spectrum quotient yields a neural mechanism for altered perception. Brain 133: 2089–2097. 20513659 10.1093/brain/awq122

[65] Glass GV, Peckham PD, Sanders JR (1972) Consequences of failure to meet assumptions underlying the fixed effects analyses of variance and covariance. Review of Educational Research 42: 237–288.

[66] Ozonoff S, Pennington BF, Rogers SJ (1991) Executive function deficits in high-functioning autistic individuals: relationship to theory of mind. J Child Psychol Psychiatry 32: 1081–1105. 1787138 10.1111/j.1469-7610.1991.tb00351.x

[67] Ozonoff S, Rogers SJ, Pennington BF (1991) Asperger’s syndrome: evidence of an empirical distinction from high-functioning autism. J Child Psychol Psychiatry 32: 1107–1122. 1787139 10.1111/j.1469-7610.1991.tb00352.x

[68] Ozonoff S, Strayer DL (1997) Inhibitory function in nonretarded children with autism. J Autism Dev Disord 27: 59–77. 9018582 10.1023/a:1025821222046

[69] Ozonoff S (1997) Components of executive function in autism and other disorders. In: Russell J, editor. Autism as an executive disorder. New York: Oxford University Press. 179–211.

[70] Rumsey JM (1985) Conceptual problem-solving in highly verbal, nonretarded autistic men. J Autism Dev Disord 15: 23–36. 3980427 10.1007/BF01837896

[71] Rumsey JM, Hamburger SD (1988) Neuropsychological findings in high-functioning men with infantile autism, residual state. J Clin Exp Neuropsychol 10: 201–221. 3350920 10.1080/01688638808408236

[72] Hughes C, Russell J, Robbins TW (1994) Evidence for executive dysfunction in autism. Neuropsychologia 32: 477–492. 8047253 10.1016/0028-3932(94)90092-2

[73] Hill EL (2004) Evaluating the theory of executive dysfunction in autism. Developmental Review 24: 189–233.

[74] Geurts HM, Corbett B, Solomon M (2009) The paradox of cognitive flexibility in autism. Trends Cogn Sci 13: 74–82. 19138551 10.1016/j.tics.2008.11.006 PMC5538880

[75] Hill EL (2004) Executive dysfunction in autism. Trends Cogn Sci 8: 26–32. 14697400 10.1016/j.tics.2003.11.003

[76] Green DM, Swets JA (1966) Signal detection theory and psychophysics. New York, NY: Wiley.

[77] Gathercole SE, Willis CS, Baddeley AD, Emslie H (1994) The Children’s Test of Nonword Repetition: a test of phonological working memory. Memory 2: 103–127. 7584287 10.1080/09658219408258940

[78] Tiffin J, Asher EJ (1948) The Purdue pegboard; norms and studies of reliability and validity. J Appl Psychol 32: 234–247. 18867059 10.1037/h0061266

[79] Baron-Cohen S, Ashwin E, Ashwin C, Tavassoli T, Chakrabarti B (2009) Talent in autism: hyper-systemizing, hyper-attention to detail and sensory hypersensitivity. Philos Trans R Soc Lond B Biol Sci 364: 1377–1383. 19528020 10.1098/rstb.2008.0337 PMC2677592

[80] Shah A, Frith U (1983) An islet of ability in autistic children: a research note. J Child Psychol Psychiatry 24: 613–620. 6630333 10.1111/j.1469-7610.1983.tb00137.x

[81] Jolliffe T, Baron-Cohen S (1997) Are people with autism and Asperger syndrome faster than normal on the Embedded Figures Test? J Child Psychol Psychiatry 38: 527–534. 9255696 10.1111/j.1469-7610.1997.tb01539.x

[82] Frith U (1989) Autism: Explaining the enigma. Oxford, UK: Blackwell.

[83] Happe F (1999) Autism: cognitive deficit or cognitive style? Trends Cogn Sci 3: 216–222. 10354574 10.1016/s1364-6613(99)01318-2

[84] Mottron L, Burack JA (2001) Enhanced perceptual functioning in the development of autism. In: Burack JA, Charman T, Yirmiya N, Zelazo PR, editors. The development of autism: Perspectives from theory and research. Mahwah, NJ: Lawrence Erlbaum Associates. 131–148.

[85] Mottron L, Dawson M, Soulieres I, Hubert B, Burack J (2006) Enhanced perceptual functioning in autism: an update, and eight principles of autistic perception. J Autism Dev Disord 36: 27–43. 16453071 10.1007/s10803-005-0040-7

[86] Plaisted K, O’Riordan M, Baron-Cohen S (1998) Enhanced discrimination of novel, highly similar stimuli by adults with autism during a perceptual learning task. J Child Psychol Psychiatry 39: 765–775. 9690939

[87] Plaisted K, O’Riordan M, Baron-Cohen S (1998) Enhanced visual search for a conjunctive target in autism: a research note. J Child Psychol Psychiatry 39: 777–783. 9690940

[88] Witkin H, Oltman P, Raskin E, Karp S (1971) A manual for the Embedded Figures Test. Mountain View, CA: Consulting Psychologists Press, Inc.

[89] White SJ, Saldana D (2011) Performance of children with autism on the Embedded Figures Test: a closer look at a popular task. J Autism Dev Disord 41: 1565–1572. 21350920 10.1007/s10803-011-1182-4

[90] Jarrold C, Gilchrist ID, Bender A (2005) Embedded figures detection in autism and typical development: preliminary evidence of a double dissociation in relationships with visual search. Dev Sci 8: 344–351. 15985068 10.1111/j.1467-7687.2005.00422.x

[91] Kaland N, Mortensen EL, Smith L (2007) Disembedding performance in children and adolescents with Asperger syndrome or high-functioning autism. Autism 11: 81–92. 17175576 10.1177/1362361307070988

[92] Ropar D, Mitchell P (2001) Susceptibility to illusions and performance on visuospatial tasks in individuals with autism. J Child Psychol Psychiatry 42: 539–549. 11383970

[93] Asperger H (1944) Die “autistichen psychopathen” im kindersalter [Autistic psychopathy in childhood]. Archive fur Psychiatrie und Nervenkrankheiten 117: 76–136.

[94] Jansiewicz EM, Goldberg MC, Newschaffer CJ, Denckla MB, Landa R, et al (2006) Motor signs distinguish children with high functioning autism and Asperger’s syndrome from controls. J Autism Dev Disord 36: 613–621. 16609826 10.1007/s10803-006-0109-y

[95] Green D, Charman T, Pickles A, Chandler S, Loucas T, et al (2009) Impairment in movement skills of children with autistic spectrum disorders. Dev Med Child Neurol 51: 311–316. 19207298 10.1111/j.1469-8749.2008.03242.x

[96] Mari M, Castiello U, Marks D, Marraffa C, Prior M (2003) The reach-to-grasp movement in children with autism spectrum disorder. Philos Trans R Soc Lond B Biol Sci 358: 393–403. 12639336 10.1098/rstb.2002.1205 PMC1693116

[97] Pan CY, Tsai CL, Chu CH (2009) Fundamental movement skills in children diagnosed with autism spectrum disorders and attention deficit hyperactivity disorder. J Autism Dev Disord 39: 1694–1705. 19588236 10.1007/s10803-009-0813-5

[98] Provost B, Lopez BR, Heimerl S (2007) A comparison of motor delays in young children: autism spectrum disorder, developmental delay, and developmental concerns. J Autism Dev Disord 37: 321–328. 16868847 10.1007/s10803-006-0170-6

[99] Hilton CL, Zhang Y, White MR, Klohr CL, Constantino J (2012) Motor Impairment in Sibling Pairs Concordant and Discordant for Autism Spectrum Disorders. Autism 16: 430–441. 22013131 10.1177/1362361311423018 PMC4222044

[100] MacNeil LK, Mostofsky SH (2012) Specificity of dyspraxia in children with autism. Neuropsychology 26: 165–171. 22288405 10.1037/a0026955 PMC3312580

[101] Dowell LR, Mahone EM, Mostofsky SH (2009) Associations of postural knowledge and basic motor skill with dyspraxia in autism: implication for abnormalities in distributed connectivity and motor learning. Neuropsychology 23: 563–570. 19702410 10.1037/a0015640 PMC2740626

[102] Fournier KA, Hass CJ, Naik SK, Lodha N, Cauraugh JH (2010) Motor coordination in autism spectrum disorders: a synthesis and meta-analysis. J Autism Dev Disord 40: 1227–1240. 20195737 10.1007/s10803-010-0981-3

[103] Burack JA, Iarocci G, Bowler D, Mottron L (2002) Benefits and pitfalls in the merging of disciplines: the example of developmental psychopathology and the study of persons with autism. Dev Psychopathol 14: 225–237. 12030689 10.1017/s095457940200202x

[104] Miller GA, Chapman JP (2001) Misunderstanding analysis of covariance. J Abnorm Psychol 110: 40–48. 11261398 10.1037//0021-843x.110.1.40

[105] American Psychiatric Association (2011) DSM-5 Development: Proposed Revision- Autism Spectrum Disorder. American Psychiatric Association.

[106] Attwood T (2007) The complete guide to Asperger’s syndrome. London, UK: Jessica Kingsley Publishers.

[107] Lombardo MV, Barnes JL, Wheelwright SJ, Baron-Cohen S (2007) Self-referential cognition and empathy in autism. PLoS ONE 2: e883. 17849012 10.1371/journal.pone.0000883 PMC1964804

[108] Jones CR, Pickles A, Falcaro M, Marsden AJ, Happe F, et al (2011) A multimodal approach to emotion recognition ability in autism spectrum disorders. J Child Psychol Psychiatry 52: 275–285. 20955187 10.1111/j.1469-7610.2010.02328.x

[109] Tracy JL, Robins RW, Schriber RA, Solomon M (2011) Is emotion recognition impaired in individuals with autism spectrum disorders? J Autism Dev Disord 41: 102–109. 20464465 10.1007/s10803-010-1030-y PMC3005106

[110] Neumann D, Spezio ML, Piven J, Adolphs R (2006) Looking you in the mouth: abnormal gaze in autism resulting from impaired top-down modulation of visual attention. Soc Cogn Affect Neurosci 1: 194–202. 18985106 10.1093/scan/nsl030 PMC2555425

[111] Rutherford MD, Towns AM (2008) Scan path differences and similarities during emotion perception in those with and without autism spectrum disorders. J Autism Dev Disord 38: 1371–1381. 18297386 10.1007/s10803-007-0525-7

[112] Golarai G, Grill-Spector K, Reiss AL (2006) Autism and the development of face processing. Clin Neurosci Res 6: 145–160. 18176635 10.1016/j.cnr.2006.08.001 PMC2174902

[113] Schultz RT (2005) Developmental deficits in social perception in autism: the role of the amygdala and fusiform face area. Int J Dev Neurosci 23: 125–141. 15749240 10.1016/j.ijdevneu.2004.12.012

[114] Schultz RT, Gauthier I, Klin A, Fulbright RK, Anderson AW, et al (2000) Abnormal ventral temporal cortical activity during face discrimination among individuals with autism and Asperger syndrome. Arch Gen Psychiatry 57: 331–340. 10768694 10.1001/archpsyc.57.4.331

[115] Schultz RT, Grelotti DJ, Klin A, Kleinman J, Van der Gaag C, et al (2003) The role of the fusiform face area in social cognition: implications for the pathobiology of autism. Philos Trans R Soc Lond B Biol Sci 358: 415–427. 12639338 10.1098/rstb.2002.1208 PMC1693125

[116] Davis G, Plaisted K (2007) Autism: not interested or not ‘tuned-in’? Curr Biol 17: R851–853. 17925216 10.1016/j.cub.2007.08.003

[117] O’Hearn K, Schroer E, Minshew N, Luna B (2010) Lack of developmental improvement on a face memory task during adolescence in autism. Neuropsychologia 48: 3955–3960. 20813119 10.1016/j.neuropsychologia.2010.08.024 PMC2975893

[118] Hernandez N, Metzger A, Magne R, Bonnet-Brilhault F, Roux S, et al (2009) Exploration of core features of a human face by healthy and autistic adults analyzed by visual scanning. Neuropsychologia 47: 1004–1012. 19027761 10.1016/j.neuropsychologia.2008.10.023

[119] Sasson NJ (2006) The development of face processing in autism. J Autism Dev Disord 36: 381–394. 16572261 10.1007/s10803-006-0076-3

[120] Weigelt S, Koldewyn K, Kanwisher N (2012) Face identity recognition in autism spectrum disorders: A review of behavioral studies. Neurosci Biobehav Rev 36: 1060–1084. 22212588 10.1016/j.neubiorev.2011.12.008

[121] Krebs JF, Biswas A, Pascalis O, Kamp-Becker I, Remschmidt H, et al (2011) Face processing in children with autism spectrum disorder: independent or interactive processing of facial identity and facial expression? J Autism Dev Disord 41: 796–804. 20839043 10.1007/s10803-010-1098-4

[122] Harms MB, Martin A, Wallace GL (2010) Facial emotion recognition in autism spectrum disorders: a review of behavioral and neuroimaging studies. Neuropsychol Rev 20: 290–322. 20809200 10.1007/s11065-010-9138-6

[123] Baron-Cohen S, Ring HA, Bullmore ET, Wheelwright S, Ashwin C, et al (2000) The amygdala theory of autism. Neurosci Biobehav Rev 24: 355–364. 10781695 10.1016/s0149-7634(00)00011-7

[124] Ashwin C, Baron-Cohen S, Wheelwright S, O’Riordan M, Bullmore ET (2007) Differential activation of the amygdala and the ‘social brain’ during fearful face-processing in Asperger Syndrome. Neuropsychologia 45: 2–14. 16806312 10.1016/j.neuropsychologia.2006.04.014

[125] Adolphs R, Gosselin F, Buchanan TW, Tranel D, Schyns P, et al (2005) A mechanism for impaired fear recognition after amygdala damage. Nature 433: 68–72. 15635411 10.1038/nature03086

[126] Klin A, Jones W, Schultz R, Volkmar F, Cohen D (2002) Visual fixation patterns during viewing of naturalistic social situations as predictors of social competence in individuals with autism. Arch Gen Psychiatry 59: 809–816. 12215080 10.1001/archpsyc.59.9.809

[127] Loucas T, Riches NG, Charman T, Pickles A, Simonoff E, et al (2010) Speech perception and phonological short-term memory capacity in language impairment: preliminary evidence from adolescents with specific language impairment (SLI) and autism spectrum disorders (ASD). Int J Lang Commun Disord 45: 275–286. 20131963 10.3109/13682820902936433

[128] Turner MA (1999) Generating novel ideas: fluency performance in high-functioning and learning disabled individuals with autism. J Child Psychol Psychiatry 40: 189–201. 10188701

[129] Voyer D, Voyer S, Bryden MP (1995) Magnitude of sex differences in spatial abilities: a meta-analysis and consideration of critical variables. Psychol Bull 117: 250–270. 7724690 10.1037/0033-2909.117.2.250

[130] Jeste SS (2011) The neurology of autism spectrum disorders. Curr Opin Neurol 24: 132–139. 21293268 10.1097/WCO.0b013e3283446450 PMC3160764

[131] Nicholson KG, Kimura D (1996) Sex differences for speech and manual skill. Percept Mot Skills 82: 3–13. 8668494 10.2466/pms.1996.82.1.3

[132] Lord C, Schopler E (1985) Differences in sex ratios in autism as a function of measured intelligence. J Autism Dev Disord 15: 185–193. 3997745 10.1007/BF01531604

[133] Lord C, Schopler E, Revicki D (1982) Sex differences in autism. J Autism Dev Disord 12: 317–330. 7161234 10.1007/BF01538320

[134] Tsai LY, Stewart MA, August G (1981) Implication of sex differences in the familial transmission of infantile autism. J Autism Dev Disord 11: 165–173. 6927702 10.1007/BF01531682

[135] Wing L (1981) Sex ratios in early childhood autism and related conditions. Psychiatry Res 5: 129–137. 6945608 10.1016/0165-1781(81)90043-3

[136] Plaisted K, Swettenham J, Rees L (1999) Children with autism show local precedence in a divided attention task and global precedence in a selective attention task. J Child Psychol Psychiatry 40: 733–742. 10433407

[137] Hines M (2010) Sex-related variation in human behavior and the brain. Trends Cogn Sci 14: 448–456. 20724210 10.1016/j.tics.2010.07.005 PMC2951011

