# Supplementary material for: Cognition in Males and Females with Autism: Similarities and Differences
Source: PLoS One. 2012 Oct 17;7(10):e47198. doi: 10.1371/journal.pone.0047198 (PMC3474800; doi:10.1371/journal.pone.0047198)
Supplement: Table S1 — A brief summary of the descriptive statistics for raw scores in all the cognitive tasks. (DOC) [file pone.0047198.s001.doc]

***Supporting Information***

***Table S1.***

|  | **Male Control** | **Male ASC** | **Female Control** | **Female ASC** |
| --- | --- | --- | --- | --- |
| ***Task Scores*** | ***Mean (Standard Deviation)*** | | | |
| **Eyes Test** |  |  |  |  |
| Correct score | 26.72 (3.49) | 22.47 (5.86) | 28.47 (2.57) | 22.59 (6.82) |
| RT (ms) | 6751 (1620) | 7390 (2644) | 6154 (1726) | 7049 (2225) |
| **KDEF Test RT (ms)** |  |  |  |  |
| Happy | 1595 (345) | 2124 (657) | 1530 (344) | 2031 (784) |
| Sad | 2225 (424) | 2851 (1048) | 2100 (636) | 2536 (929) |
| Angry | 2640 (577) | 3347 (1688) | 2435 (459) | 2852 (1022) |
| Fear | 3855 (1364) | 4632 (1836) | 3442 (861) | 4650 (3242) |
| Disgusted | 2681 (639) | 3534 (1310) | 2424 (443) | 2700 (883) |
| Surprised | 2120 (492) | 3181 (1780) | 2088 (572) | 2740 (1490) |
| Neutral | 2173 (512) | 2998 (1335) | 2313 (638) | 2628 (1070) |
| **Go/No-Go** |  |  |  |  |
| ‘Go’ RT (ms) | 426 (41) | 473 (68) | 433 (48) | 453 (75) |
| SDT *d’* | 4.214 (0.769) | 3.541 (0.693) | 4.301 (0.985) | 3.686 (1.127) |
| SDT *C* | -0.039 (0.274) | -0.017 (0.196) | -0.053 (0.262) | 0.022 (0.227) |
| **F-A-S** | 44.94 (13.69) | 44.59 (13.57) | 43.16 (9.94) | 37.50 (15.14) |
| **NWR** | 21.47 (3.10) | 20.53 (3.47) | 22.19 (2.43) | 21.31 (3.27) |
| **EFT** |  |  |  |  |
| RT (all) (s) | 26.35 (15.29) | 43.93 (32.44) | 43.05 (23.25) | 52.88 (31.50) |
| RT (correct) (s) | 14.70 (8.11) | 17.09 (9.71) | 17.56 (11.38) | 21.84 (15.82) |
| **Pegboard** |  |  |  |  |
| Right-hand | 13.94 (2.36) | 12.56 (2.46) | 14.12 (1.90) | 12.97 (2.52) |
| Left-hand | 13.53 (2.31) | 12.41 (2.77) | 13.31 (1.91) | 12.09 (2.36) |
| Both-hands | 14.66 (2.31) | 15.41 (4.98) | 14.31 (2.13) | 13.47 (2.95) |
| Assembly | 36.94 (7.35) | 28.31 (8.59) | 25.50 (4.30) | 23.03 (5.61) |
